# The “Sound of Silence” in a Neonatal Intensive Care Unit—Listening to Speech and Music Inside an Incubator

**DOI:** 10.3389/fpsyg.2020.01055

**Published:** 2020-05-26

**Authors:** Matthias Bertsch, Christoph Reuter, Isabella Czedik-Eysenberg, Angelika Berger, Monika Olischar, Lisa Bartha-Doering, Vito Giordano

**Affiliations:** ^1^Department of Music Physiology, University of Music and Performing Arts Vienna, Vienna, Austria; ^2^Musicological Department, University of Vienna, Vienna, Austria; ^3^Department of Pediatrics and Adolescent Medicine, Division of Neonatology, Pediatric Intensive Care and Neuropediatrics, Comprehensive Center for Pediatrics, Medical University of Vienna, Vienna, Austria

**Keywords:** incubator, NICU, noise, music, preterm, transfer spectrum, recordings

## Abstract

**Background:** The intrauterine hearing experience differs from the extrauterine hearing exposure within a neonatal intensive care unit (NICU) setting. Also, the listening experience of a neonate drastically differs from that of an adult. Several studies have documented that the sound level within a NICU exceeds the recommended threshold by far, possibly related to hearing loss thereafter. The aim of this study was, first, to precisely define the dynamics of sounds within an incubator and, second, to give clinicians and caregivers an idea about what can be heard “inside the box.”

**Methods:** Audio recordings within an incubator were conducted at the Pediatric Simulation Center of the Medical University Vienna. They contained recorded music, speech, and synthesized sounds. To understand the dynamics of sounds around and within the incubator, the following stimuli were used: broadband noise with decreasing sound level in 10 steps of 6 dB, sine waves (62.5, 125, 250, 500, 1000, 2000, 4000, 8000, and 16,000 Hz), logarithmic sweep (Chirp) over the frequency band 20 Hz to 21 kHz, singing male voice, singing, and whispering female voice.

**Results:** Our results confirm a protective effect of the incubator from noises above 500 Hz in conditions of “no-flow” and show almost no protective effect of an incubator cover. We, furthermore, observed a strong boost of low frequencies below 125 Hz within the incubator, as well as a notable increase of higher frequency noises with open access doors, a significant resonant effect of the incubator, and a considerable masking effect of the respiratory support against any other source of noise or sound stimulation even for “low-flow” conditions.

**Conclusion:** Our study reveals high noise levels of air supply at high flow rates and the boost of low frequencies within the incubator. Education of medical staff and family members as well as modifications of the physical environment should aim at reducing noise exposure of preterm infants in the incubator. Audiovisual material is provided as Supplementary Material.

## Introduction

According to the World Health Organization (WHO), about 15 million babies are born premature every year with a ratio varying between 5 and 18% depending on the country of origin. Even if preterm birth still represents one of the most important causes of death for children younger than 5 years of age, improvements in neonatal intensive care importantly reduced mortality also among those infants weighing less than 1500 g (Liu, 2010). Nonetheless, many survivors face lifelong disabilities including visual, hearing, and cognitive impairments (Liu, 2010).

Premature babies are constantly exposed to light and noise, and even if the American Academy of Pediatrics (AAP) (1997) suggests that the noise level should be less than or equal to 45 dB during the day and 35 dB during the night (American Academy of Pediatrics (AAP), 1997, p. 724), several studies have shown that the noise level within a neonatal intensive care unit (NICU) is much higher than recommended (Bess et al., 1979; Surenthiran et al., 2003; Altuncu et al., 2009; Berg et al., 2010; Matook et al., 2010; Milette, 2010; Salandin et al., 2011).

The listening experience of a neonate differs drastically from that of an adult; however, even if immature, hearing is established as a functional sensation at the beginning of the third trimester of life (Birnholz and Benacerraf, 1983).

The intrauterine hearing experience varies severely from the extrauterine hearing exposure in the NICU setting. In particular, mainly low frequency noises (below 500 Hz) are being transmitted through the mother’s womb, while the NICU environment consists of multiple high frequency noises that exceeding recommended values for neonates. Surveillance monitors may increase the basic sound pressure level to 57 dB, and during medical visits, peaks of 82–114 and 117 dB may occur by simply opening or closing incubator doors or by the conversation between staff members (DePaul and Chambers, 1995; Marik et al., 2012; Philbin et al., 2017). Assume that these high sound levels may contribute to hearing damage or even hearing loss as diagnosed in 2–10% of preterm infants vs 0.1% of the general pediatric population (Wroblewska-Seniuk et al., 2017). Once identified in the newborn, hearing deficits have been largely located in the inner and outer hair cells within the cochlea, a region of the ear most affected by low-frequency sound (<250 Hz) (Abrams and Gerhardt, 2000). The absence of filtration and padding of sounds usually provided by the uterus can alter the postnatal maturation of the external and middle ear and the way sound is absorbed, processed, filtered, and transmitted to the auditory system (Abdala and Keefe, 2012).

Studies have shown that exposure to intense and sustained sound outside the dB and frequency range as normally heard by the fetus is harmful and may be related to stress responses, alteration in physiological stability, sleep deprivation, autonomic changes, alteration in endocrine and metabolic response, and hearing deficits (Krueger et al., 2005; Milette, 2010).

Preterm babies are usually protected from the external environment by the incubator. The incubator is a closed medical device that creates a modified environment, intended to accommodate infants born preterm or underweight, ensuring them the right temperature and humidity. Inside the incubator, the preterm is, on the one hand isolated from the acoustic world outside and, on the other hand exposed to additional noises generated internally by medical devices such as respiratory support devices (Fricke, 1993).

Many studies have examined and monitored sources of noise within different NICU settings and over a prolonged period of time (Falk and Farmer, 1973; Berg et al., 2010). Findings from these studies were essential for the detection of noise-generating factors and were further used to develop the so-called goal-driven Noise Awareness Educational Program (NAEP), also recognized by the Newborn Individualized Developmental Care and Assessment Program (NIDCAP).

Fewer studies have concentrated on understanding the dynamics of sounds within an incubator, a special environment, with caregivers, health-care providers, and also recently music therapists communicating with the premature infant through open access doors. Understanding the way sound propagates through and within an incubator could shed light and bring awareness on how to communicate/interact with the premature infant, on how to re-organize the NICU setting, and on how to ameliorate environmental sounds like device’s alarms.

Therefore, the aim of this study was to precisely examine the dynamics of sounds within the incubator. Besides gaining important information about the auditory stimulation of the neonate lying in the incubator, this study was thought to provide detailed information and listening examples of what can be really heard from “inside the box.”

We wanted to produce and share educational sound material to catch the attention of clinicians, caretakers, parents, and music therapists and to show how sound information from the external environment is perceived from inside the incubator, the way incubators can protect from different noise levels at different frequencies, and the way incubators amplify and modulate certain sounds on the other hand.

## Materials and Methods

In an empirical study, audio recordings were conducted inside and outside of an incubator. These recordings provided the basis for the analysis of the transfer function and enabled to provide supplementary acoustic demonstration material.

### Setting

Reference microphones were tested within the anechoic chamber of the University of Music and Performing Arts Vienna, while the experimental recordings were done at the Center for Pediatric Simulation Training of the Medical University Vienna.

### Material and Procedure

For calibration purposes of the recording equipment, the loudspeaker (Mackie MR624) and microphones (Esper K4) were measured. The monitor box Mackie MR624, with a 6,5” woofer und 1” tweeter has a frequency response of ± 3 dB from 45 Hz to 20 kHz and delivers 40 watts (4 Ω load). Settings were Space = A, U = Normal, EQ = 0 dB. The paired 1/2” microphones Esper K4 were calibrated, resulting in a similar characteristic with a variation of 2–3 dB.

Sound signals were played to the active speakers and simultaneously recorded through an external USB audio interface Steinberg UR22, with 44.1 kHz/16 bit using the audio software Audacity (Version 2.3.0 by Dominic Mazzoni and the Audacity Team) on a MacBook Pro (MacBook Pro, Apple Inc.).

For the recording session in the simulation room, we used the incubator Dräger Isolette^®^ Infant Incubator C2000 in a usual position 123 cm above the floor. The Dräger Isolette^®^ Infant Incubator (Model C2000) has a dimension of 102.9 cm width and 67.3 cm depth, and the mattress tray has a width of 79 cm and 41 cm depth. Temperature inside was controlled for 26°C. According to the incubator’s manual, the noise level inside the incubator is less than 47 dB(A) when located in a room with a noise floor level of less than 37 dB(A). The incubator was placed in a room with a perimeter of 6.5 m × 3.5 m with a height of 2.4 m.

**FIGURE 1:**
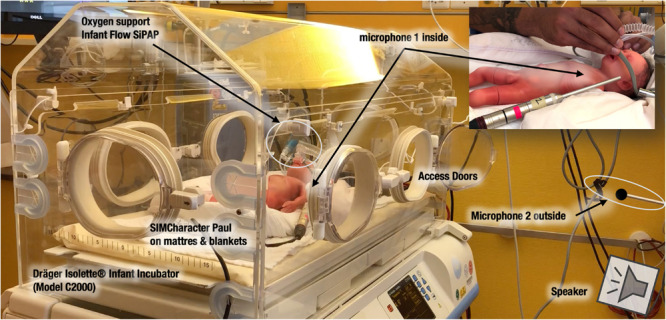
Setup of the recording session in the simulation room with a Dräger Isolette Infant Incubator C2000 in the Center for Pediatric Simulation Training of the Medical University Vienna. Microphones are positioned inside and outside the “box.” On the top right, one can see the microphone 2 inside, near the ear of the SIMCharacter Paul from another point of view.

Nobody was present in the room during the audio recording session. As a patient model for this study, a preterm manekin was used (Paul, SIMCharacters^®^ GmbH, Vienna, Austria). Paul has a size of 35 cm, weighs less than 1000 g, and is the most accurate representation of a preterm infant born at 27 weeks of gestation. Paul received oxygen support through the Infant Flow^®^ SiPAP system by CareFusion (Vyaire Medical). The microphone inside the incubator was positioned 4 cm next to the left ear of Paul, and 4 cm above a sheet. The speaker outside the incubator was placed at a distance of 105 cm from the incubator. Additional microphones (e.g., an artificial head) have been positioned but not used for this study (Figure 1).

For a measurement of the noise floor level, an NTI Audio XL2 SPL meter was used.

### Sound Stimuli

The sound file used as a source signal has a total duration of 9 min. It contains recorded music, speech, and synthesized sounds, either created in Audacity or imported from the sound example library of the Acoustical Society of America (ASA, 1987). The source signal has the same PCM wave quality (16 bit, 44.1 Hz sampling rate) as the recordings. To understand the dynamics of sounds around and within the incubator the following stimuli were used:

– Acoustic stimulus 1: Broadband noise with decreasing sound level in 10 steps of 6 dB.
– Acoustic stimulus 2: Sine wave signals at different frequencies in ten decreasing steps of 5 dB each (125, 250, 500, 1000, 2000, 4000, and 8000 Hz).
– Acoustic stimulus 3: Sine wave signals (62.5, 125, 250, 500, 1000, 2000, 4000, 8000, and 16,000 Hz).
– Acoustic stimulus 4: Logarithmic sweep (chirp) over the frequency band from 20 Hz to 21 kHz with a duration of 15 s.
– Acoustic stimulus 5: Male voice singing.
– Acoustic stimulus 6: Female voice singing and speaking.
– Acoustic stimulus 7: Female voice softly singing/whispering a children’s song.
– Acoustic stimulus 8: White noise.

Supplementary to these, further stimuli that could be part of a real-life setting have been recorded but were not included in this analysis.

### Recording Conditions

First, a reference recording using the speaker and both microphones was done in the anechoic chamber of the University of Music and Performing Arts, Vienna, Austria. Then, nine recording conditions, all using microphone 1 inside and microphone 2 outside the incubator, were conducted within the simulation room (Table 1). The incubator has been recorded with closed access doors while being covered with a blanket as well as being uncovered with closed and with opened access doors. For each of these situations (covered with doors closed, uncovered with doors closed, and uncovered with doors opened), the SiPAP flow system provided a flow of 0 (was switched off), 8 or 12 L/min.

**TABLE 1:** Recording conditions (n.a.: not applicable).

Recording	Covered with blanket	Closed doors	SiPAP flow system	Microphone	Supplementary audio filename	Position
*n.a. (source)*	*n.a.*	*n.a.*	*n.a.*	*digital*	*SOURCE (original digital audio)*	*n.a.*
Reference 1	n.a.	n.a.	n.a.	1	REF 01 (anechoic chamber mic1)	Anechoic chamber
Reference 2	n.a.	n.a.	n.a.	2	REF 02 (anechoic chamber mic2)	Anechoic chamber
0	Yes	Yes	0	1	REC 00 (co cl 00 mic1 outside)	Outside incubator
1	Yes	Yes	0	2	REC 01 (co cl 00 mic2 inside)	Inside the incubator
2	Yes	Yes	8	2	REC 02 (co cl 08 mic2 inside)	Inside the incubator
3	Yes	Yes	12	2	REC 03 (co cl 12 mic2 inside)	Inside the incubator
4	No	Yes	0	2	REC 04 (uc cl 00 mic2 inside)	Inside the incubator
5	No	Yes	8	2	REC 05 (uc cl 08 mic2 inside)	Inside the incubator
6	No	Yes	12	2	REC 06 (uc cl 12 mic2 inside)	Inside the incubator
7	No	No	0	2	REC 07 (uc op 00 mic2 inside)	Inside the incubator
8	No	No	8	2	REC 08 (uc op 08 mic2 inside)	Inside the incubator
9	No	No	12	2	REC 09 (uc op 12 mic2 inside)	Inside the incubator

### Sound Analysis

In order to compare the influence of the different environmental conditions (inside/outside incubator, doors open/closed, use of covering blanket, different flow settings) on the sound of the stimuli, the recorded signals (8 stimuli × 11 conditions) were analyzed and visualized with regard to their signal-to-noise ratio (SNR) as well as their intensity in different frequency bands.

**FIGURE 2:**
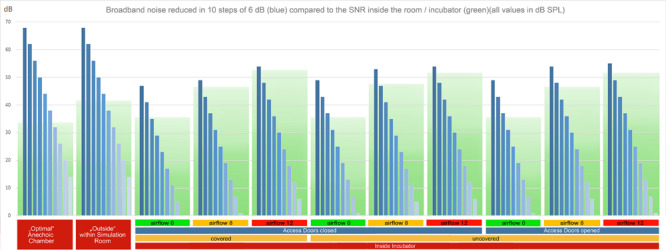
RMS levels of broadband noise with decreasing sound volume in ten steps of 6 dB (blue bars) in reference to the noise floor (green boxes) for all recording setups. The lower and dark green part of the boxes represents the masking threshold. Noises with intensities (blue bars) above the green masking floor are audible without masking. In cases with higher flow rates, a high proportion of the stimuli are masked (Supplementary Audio Material, Acoustic Stimulus 1).

**FIGURE 3:**
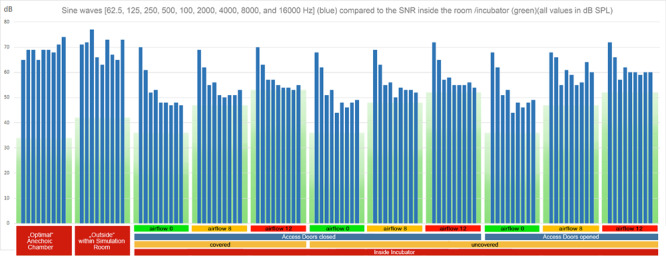
RMS levels of sine waves (62.5, 125, 250, 500, 1000, 2000, 4000, 8000, and 16,000 Hz) (blue bars) in reference to the noise floor (green boxes, with the lower part in darker green representing the masking threshold) for all recording setups. As in Figure 2, only pure tones with intensities (blue bars) above the green masking floor are audible. In cases with higher flow rates, a high proportion of the stimuli are masked (Supplementary Audio Material, Acoustic Stimulus 2).

In order to examine the SNR as well as the masking threshold outside and inside the incubator under different conditions, we measured the noise floor level inside and outside of the incubator (inside, covered with a blanket, doors closed, no air flow: 36 dB SPL, outside: 42 dB SPL). Using these values as reference values, we calculated the noise floor levels of the other conditions (the noise floor increases with the air flow). To visualize the broadband noise decreasing in 6 dB steps under each condition (Figure 2), the respective RMS sound level of the strongest noise has been determined based on the respective noise floor level. From this maximum sound level, the decreasing 6 dB steps have been added visualizing the disappearing broadband noise below the masking threshold. In case of the visualization of the pure tones (Figure 3), their sound levels have been determined in the same way based on the respective noise floor level of each recording.

**FIGURE 4:**
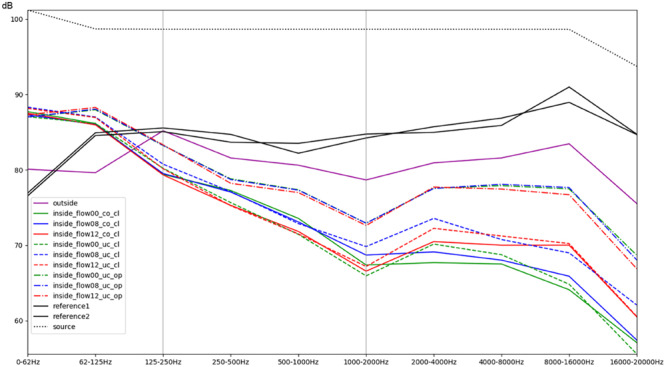
Comparison of different conditions with regards to the mean RMS energy in octave bands for a sweep sound (stimulus 4). The colors green, blue, and red correspond to increasing intensity of respiratory flow (flow00 = without any flow, flow08 = medium flow of 8 L/min, flow12 = high flow of 12 L/min). Dashed lines indicate whether the box was uncovered (uc) or covered with a blanket (co), as well as whether the doors were closed (cl) or opened (op). The purple line represents the recordings outside of the incubator inside the simulation room; the black lines correspond to the reference recordings of both microphones within the anechoic chamber (Supplementary Audio Material, Acoustic Stimulus 4).

**FIGURE 5:**
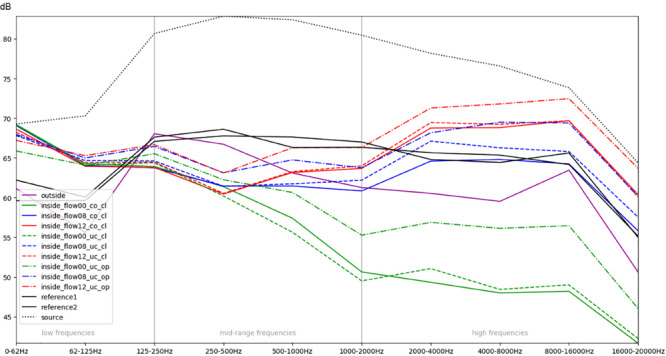
Comparison of different conditions (aggregated across all eight stimuli each) with regard to the mean RMS energy in different frequency bands.

**FIGURE 6:**
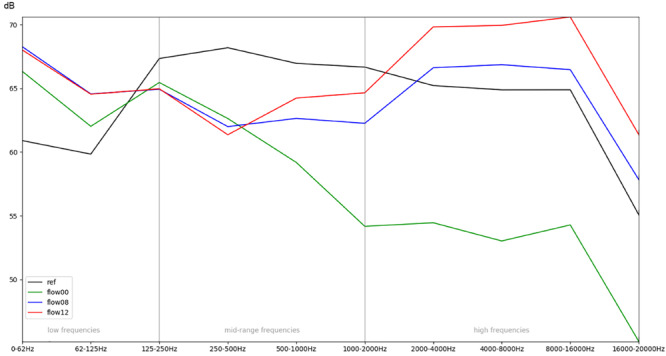
Comparison of the recordings inside the incubator, while different intensities of respiratory flow (flow00 = no flow, flow08 = medium flow, flow12 = high flow) are active.

**FIGURE 7:**
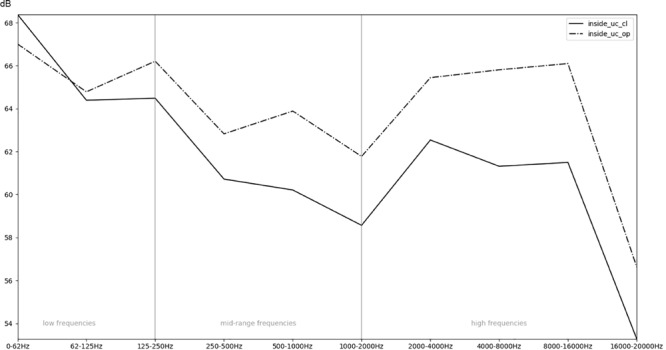
Comparison of the recordings inside an incubator while the doors are open (op) or closed (cl).

**FIGURE 8:**
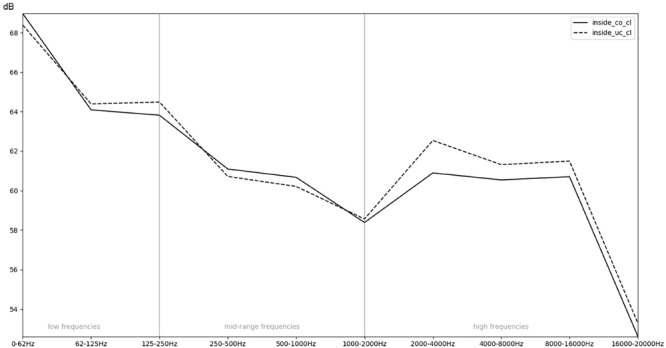
Comparison of the recordings inside an uncovered (un) or covered (co) incubator with regards to the mean RMS energy in different frequency bands.

**FIGURE 9:**
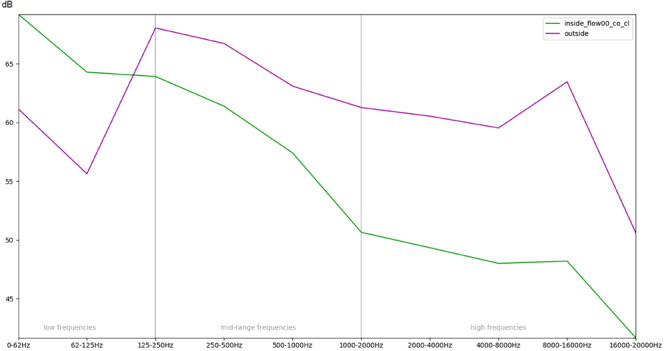
Comparison of the recordings outside of the incubator and inside a covered (co) incubator with closed doors (cl) and no respiratory flow with regards to the mean RMS energy in different frequency bands.

In order to further illustrate the influence of the environmental conditions on the sound experience, Figures 4–9 present resulting differences at the level of individual frequency bands. To this end, each signal was decomposed into ten octave bands using non-overlapping elliptic band-pass filters with cut-off frequencies of 62, 125, 250, 500, 1000, 2000, 4000, 8000, and 16,000 Hz. This was performed in MATLAB, using a filter implemented in MIRtoolbox (Lartillot et al., 2008). For each of these band-pass signals, the root-mean-square (RMS) energy was calculated and converted to a decibel value. The reference value for decibel conversion was chosen in accordance with the dB (SPL) value that was measured using the sound level meter for a test sound. Aggregated over all stimuli (except for Figure 4 which focuses on the sweep stimulus), the mean level was then calculated for each sub-band depending on the conditions to compare. Results were visualized using matplotlib in Python. Figures 4, 5 provide an overview of this comparison for all individual conditions. In Figure 6, the effects of different intensities of respiratory flow were compared (no flow, medium flow, high flow). The impact of whether the access doors of the incubator are closed or opened is illustrated in Figure 7, while Figure 8 focuses on the effects of covering the box using a blanket supposing that the incubator doors remain closed. Data are aggregated across different flow intensities in the latter two. The differences between the sound experience outside of the incubator and inside a covered incubator with closed doors and no flow activated is illustrated in Figure 9. Sound levels for routine medical actions such as opening or closing the incubator doors were also descriptively documented. Finally, to detect statistical differences in different band frequencies, a *t*-test was conducted across all stimuli for the most important conditions to compare noise level outside vs inside the incubator, the protective effect of the cover, and the impact of air-flow support within the incubator (Table 1).

## Results

### Analysis of Data

#### Acoustic Stimulus 1: Broadband Noise With Decreasing Sound Level in 10 Steps of 6 dB

The digital source sample of the acoustic stimulus 1 had a range of 60 dB from the strongest to the weakest broadband noise and an SNR above 60 dB. Limitations of speaker and microphone lead to a decrease of the SNR to less than 48 dB in the reference recording within the anechoic chamber (cf. Figure 2). Within the simulation room, the SNR decreased below 42 dB outside the incubator (cf. Figure 2), and below 36 dB inside a covered incubator with closed doors (cf. Figure 2). In general, the increased levels of the low frequencies (62.5 and 125 Hz) compared to the other frequency bands displayed an intrinsic resonance component of the incubator *per se*. Within the covered incubator with closed doors, the flow level of the SiPAP flow system (at 8 and 12 L/min) dramatically increased the noise floor level. In general, the SNR was larger in recording situations with access doors opened (Rec. 7, 8, and 9). The consistent acoustic difference can be heard in the acoustic example 1 (Supplementary Audio Material S1). While listening to the “stimulus 1 SOURCE (original digital audio),” 10 steps of decreasing intensities can be deciphered. About six to seven steps of decreased intensity can be heard in “stimulus 1 REF 02 (anechoic chamber-mic2),” while only five to six steps at “stimulus 1 REC 01 (co-cl-00-mic2-inside)” and three steps at “stimulus 1 REC 01 (co-cl-00-mic2-inside)” can be recognized. The masking effect of the airflow is visualized in Figure 2 through a green masking area behind the blue dB values.

#### Acoustic Stimulus 2: Pure Tones (62.5, 125, 250, 500, 1000, 2000, 4000, 8000, and 16,000 Hz)

The original source sample of the acoustic stimulus 2 (Supplementary Audio Material S2) consisted of a row of pure tones with equal intensity each an octave apart (62.5, 125, 250, 500, 1000, 2000, 4000, 8000, and 16,000 Hz). The reference recording showed about 7 dB differences in the recording level, with about 3 dB less intensity at 62 and 500 Hz, and a slight increase above 1000 Hz. These small deviations from equal intensity were probably caused by the recording setup of speaker and microphone. The recording outside the incubator within the simulated NICU setting was clearly influenced by standing resonance waves generated within the room with variations up to 20 dB (Figure 3). Recordings within the incubator showed a strong attenuation of all except low frequencies at 62.5 and 125 Hz (Figure 3). The sound characteristics were similar when the access doors were closed (Figure 3). With access doors opened, the lowest frequencies still dominated the spectral quality, but especially higher frequencies were notably less attenuated (Figure 3).

#### Acoustic Stimulus 3: Sine Wave Signals (62.5, 125, 250, 500, 1000, 2000, 4000, 8000, and 16,000 Hz)

Acoustic stimulus 3 is a shorter version of stimulus 2, with equal intensities of the sine waves, and is provided as Supplementary Audio Material S3.

#### Acoustic Stimulus 4: Logarithmic Sweep (Chirp) Over the Frequency Band 20 Hz to 21 kHz

Acoustic stimulus 4 (Acoustic Supplementary Material S4) used a logarithmic sweep (chirp) over the total hearing range from 20 to 21.000 Hz in a 15 s time span. The spectral analysis is illustrated in Figure 4. It shows a boost of lower frequencies within the incubator with an increase of about 6–8 dB of frequencies below 62 Hz and between 62 and 125 Hz (compare the purple line of the recording outside with the recordings inside without flow, represented as the solid blue, green, and red lines). Furthermore, it demonstrates a protective effect of the incubator from noises with a 5–8 dB reduction of intensity in frequencies above 500 Hz and an up to 15 dB damping of frequencies above 1000 Hz (Figure 4). A minimally protective albeit not significant effect can be observed when using the incubator cover, while an obvious increase in recorded noise levels within the incubator, particularly starting from mid-range frequencies of 250–500 Hz, can be seen when access doors were open. The increased noise ratio and much larger amplitudes for higher SiPAP flow rates are also evident (Figure 4).

#### Mean Values Over Several Stimuli

In order to obtain a more general perspective, a spectral comparison has been calculated and aggregated across all eight stimuli, including test sounds as well as more realistic real-world examples such as male and female voices singing and speaking (see section “Sound Stimuli”). In Figure 5, these mean values are visualized for each of the conditions. Figure 6, focusing on different intensities of respiratory flow, shows that an increased flow rate dramatically forces up frequencies above 1 kHz. This increase in intensity between flow rates is significant for all frequency bands starting from 500 Hz according to paired *t*-tests conducted across all stimuli (*p* < 0.01). Figure 7 demonstrates the increase of frequencies above 250 Hz when incubator doors are opened (*p* < 0.01 for all frequency bands starting from 125 Hz according to paired *t*-tests). All *p*-values with regard to different conditions considered are presented in Table 1. Furthermore, Figure 8 demonstrates the comparatively minor acoustic effect of the incubator cover (Obviously, a cover serves more importantly as a light shield or acts as a damping material for impact sound, e.g., if someone drops a key on top of the incubator).

In summary, Figure 9 illustrates the overall effect of the quietest condition, i.e., there is no flow inside the box, doors are closed, and the top is covered with a blanket, when compared with the sound outside the incubator.

#### Acoustic Stimuli 5, 6, and 7: Male Singing vs Female Singing vs Female Whispering

The following stimuli were used in order to emulate a NICU setting in which a care-giving person (mother, father, nurses, doctors, music therapist) tries to communicate with the infant when speaking/whispering or singing. According to the acoustic example (Acoustic Supplementary Materials S5–S7), the emitted sound recorded next to the preterm infants ear was extremely soft, if audible at all, when the airflow was on in all considered conditions. A SiPAP flow of 12 L/min leads to a boost effect of the noise level covering mostly the female voice while whispering. Sounds of frequencies below 200 Hz were predominant in incubators. Since the female voice was above 200 Hz, the sound level was very low inside the incubator with access doors closed. Open access doors increased the sound level of frequencies above 200 Hz by more than 15 dB.

The acoustic stimulus 6 was provided as Supplementary Material in order to provide a listening representation (Female voice singing and speaking, Acoustic Supplementary Material S6). The recording “stimulus 6 REC 00 (co-cl-00-mic1 outside)” is a realistic sound of a mother or caretaker speaking softly outside the incubator. This record “stimulus 6 REC 09 (uc-op-12-mic2-inside)” demonstrates which sounds are audible at the position of the ear of Paul (SIMCharacter preterm simulator) inside the incubator, when the access doors are open, and typical high airflow is provided.

#### Absolute Noise Levels

**FIGURE 10:**
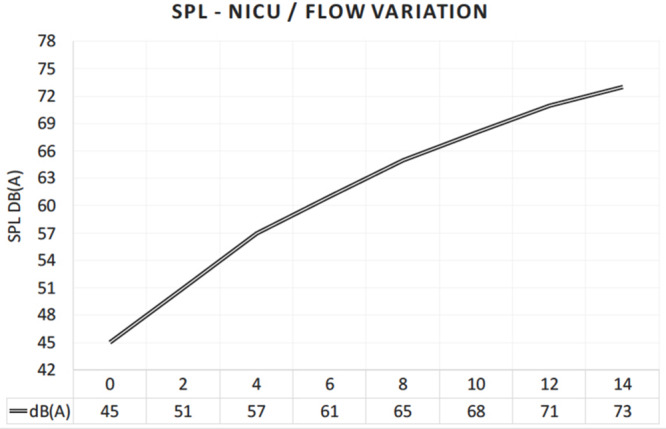
Increasing noise level inside the incubator, when the air flow of the respiratory support device (SiPAP) is continuously increased by 2 L/min.

Absolute values for routine medical actions were measured showing: (a) 62 dB outside the incubator vs 84 dB inside the incubator for the placement of a blood pressure gauge on the incubator; (b) 64 dB outside the incubator vs 85 dB inside the incubator for medical materials placed on the incubator (e.g., sterile cardboard containing material for blood sample); and (c) 73 dB outside the incubator vs 91 dB inside the incubator for closing the incubator’s door. Air-support noise values were also collected. Increasing SiPAP flow rates dramatically increased the absolute noise level. Without any flow, and in the silent room without any other sound source, a value of 45 dB(A) was measured. Figure 10 shows the increasing noise levels from 45 up to 73 dB(A) by increasing the flow rates in steps of 2 l/min. Typically, between 8 and up to 14 L/min are used to support breathing in the NICU setting. At 12 L/min, the noise level measured was around 71 dB(A) (Figure 10).

### Interactive Visualization

**FIGURE 11:**
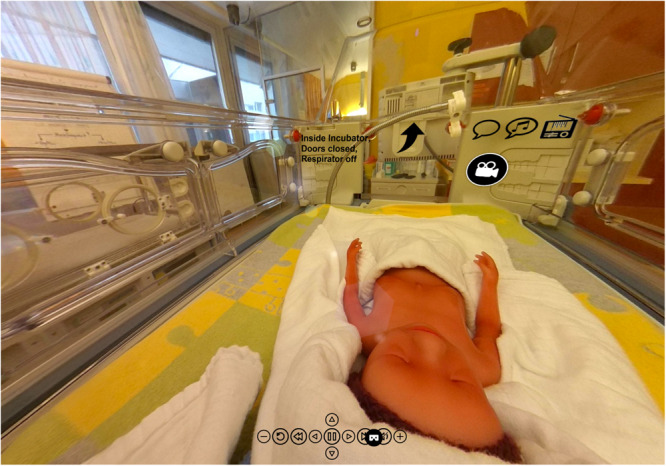
Visual information from within an incubator has been post-produced in the Center for Pediatric Simulation Training of the Medical University Vienna. The originally recorded acoustic stimuli can be heard with 360° view from inside the incubator, from the position of the SIMCharacter Paul, a preterm manikin. The video can also be seen through a 3D VR headset, providing an impressive acoustic and visual demonstration of the environment from within the incubator (Supplementary Video Material, The Incubator Experience).

Additional information about study results and acoustic examples can be found in Supplementary Video S1. The acoustic stimuli can be listened to with 360° view from inside the incubator. The video can also be watched through a 3D VR headset, providing an immersive acoustic and visual demonstration of the environment within the incubator (Figure 11).

## Discussion

The goal of this study was to provide acoustic information from outside and within the incubator typically used in a NICU. The information gained by this study is useful to understand the noise exposition of preterm infants in the NICU setting, but also provides an insight on sound’s dynamics of parents or care givers trying to communicate with infants through the incubator environment. The results of this study show the protective effect of the incubator from noises in the mid- and high frequency range in conditions of “no-flow” from a respiratory support device, the strong boost of low frequencies below 125 Hz recorded within the incubator, the notable increase of higher frequency noises with open access doors, the resonance effect of the incubator, and the considerable masking effect of the respiratory support against any other source of noise or sound stimulation even in “low-flow-level” conditions.

This study confirms previous reports on the high noise levels within an incubator, especially when respiratory support is given to the infant. We could demonstrate a tremendous increase in noise levels with increasing air-flow of the SiPAP device. In this regard, a flow reduction of 2 L/min reduced the noise level by 3 dB indicating a reduction of the sound energy by 25%. In line with these results, Surenthiran et al. (2003) measured the mean noise level in the post-natal space of preterm infants at different flow rates of a continuous positive airway pressure (CPAP) device similar to the SiPAP system, and found a noise level of 87 dB at lower frequencies (around 200–1000 Hz) at a flow rate of 5 L/min and above 101 dB at a flow rate of 10 L/min. CPAP produces a positive pressure encouraging also the opening of the Eustachian tube (Olejnik and Lehman, 2018). The regulation of pressure in the middle ear is the most important function of the Eustachian tube. Normally, the Eustachian tube is closed, but it opens during swallowing, chewing, and sneezing to equalize the middle ear pressure with atmospheric pressure (Aksoy et al., 2010). Eustachian tube dysfunction is often linked to proprioceptive/vestibular decompensating symptoms (Agrup et al., 2007). Interestingly, sensory processing disorders associated with proprioceptive abilities are often documented in preterm infants (Ryckman et al., 2017).

Olejnik and Lehman (2018) also measured sound levels under different conditions within a NICU setting. They showed that some noise sources were dependent on activities of medical-staff (such as dynamic closing of the incubator windows [85 dB]; putting a bottle on the incubator from a height of 10 cm [90 dB]; having a conversation near the incubator [55–65 dB]; dynamic closing of a drawer of the incubator [89 dB]), while others were generated by medical devices (such as conventional ventilation [38–56 dB], high flow nasal cannula, or continuous positive airways pressure [86–100 dB], or alarm peaks [85–100 dB]). Given that air support devices exceed recommendations for noise levels by far, less attention might be given on medical-staff depending actions. Similar to the findings of Olejnik and Lehman (2018), we found values between 84 and 91 dB for actions such as closing incubator doors or laying something on the top of the incubator. Considering the structure-borne sound transmission of the incubator combined with its strong resonance (especially at low frequencies), short noises produced outside the incubator walls have the potential to generate a gain of approximately 20 dB inside the incubator. Also, it is mandatory to emphasize that the above-mentioned actions carry intrinsic vibroacoustic informations that are differently filtered and elaborated within the maternal womb.

In fact, in the maternal womb, extrauterine sounds are conducted first by the mother’s amniotic fluid and then by the child’s bone system, reaching the fetal hearing organ in a very attenuated manner and not exceeding 30 dB (Olejnik and Lehman, 2018). Exposure to different vibroacoustic information, rather than only increased noise levels could be a key point to understand multisensory integration problems in children born preterm.

Furthermore, it is known that the noise level in the NICU environment frequently exceeds the recommended level over time. All studies show noise levels way above the limit proposed by the American Academy of Pediatrics (AAP) (1997) of 45 dB during daytime and 35 dB during nighttime. Berg et al. (2010) monitored sources of noise in a tertiary NICU for 24 h a day for 1 week and described noise levels exceeding the recommended threshold most of the time. The mean average noise level was 57 dB, with peaks between 82 and 102 dB, occurring during medical rounds and visiting hours, and peak noise levels around 75 dB were generated from monitors, alarms, and equipment.

Recent research from our group has shown that preterm infants exhibit significant deficits in auditory speech discrimination at term-equivalent age compared to full term neonates (Bartha-Doering et al., 2019). We hypothesized that differences in the early auditory experience of preterm vs full term infants caused these discrimination deficits. Indeed, previous studies have revealed that the preterm infant’s brain is shaped by exposure to maternal sounds in the first weeks of life and thus underline the importance of auditory experience before maturity (Webb et al., 2015). Hence, early auditory experience can have substantial impact on the structural and functional development of the auditory cortex (Chang and Merzenich, 2003; Harshaw and Lickliter, 2011). Reducing detrimental noise levels and increasing harmless sounds in the NICU may thus both improve the physiologic stability of preterm neonates as well as foster normal auditory cortex development.

Preterm infants, hospitalized in the NICU, should be stimulated with sound stimuli that affect the proper development and recovery. Alternatively, reduction of detrimental noise could be achieved by damping materials, such as pyramidal shaped acoustic foam, or by using infant earplugs (Altuncu et al., 2009). However, data on a potentially detrimental effect of noise deprivation and a potential amplification of the perceived noise level by bone conduction when using earplugs in preterm infants are missing. In any case, because of the strong resonances measured in the frequency band between 62.5 and 125 Hz, everyday noises with a large amount of low frequency energy (like noises emitted by construction sites, railways, truck transportation, ventilation, loud and bass-loaded music from television or radio equipment) should be avoided. Also, as suggested from Olejnik and Lehman (2018), the sound level should be continually monitored by means of professional devices; extra equipment surrounding the incubator should be avoided; use of incubator covers should be promoted as well as nesting and wrapping the infant in soft materials; the volume of the alarm should be reduced as much as possible.

Even if noise should be reduced to a minimum in a NICU setting, research in music therapy showed, during the last decades, promising effects as a therapeutic intervention in premature infants. Specifically, it has been shown that relaxing music therapy can reduce stress and hyper-alertness in premature infants and therefore have a positive influence on the respiratory rate, systolic and diastolic blood pressure, and heart rate (Standley, 2012; Caparros-Gonzalez et al., 2018). According to our results, therapists should be aware of the boost of lower frequencies when performing an instrument outside the incubator, and therefore should rather avoid loud bass instruments. We also suggest not to use instruments below the pitch C3, and especially instruments such as double-bass, (E-)guitars, pianos, keyboards, and brass instruments.

Importantly, reeducation devoted to the entire care team should aim at behavioral changes to decrease sound levels in the NICU (Milette, 2010). This could be achieved by referring to recommended developmental care programs such as the “NIDCAP” or the “NAEP.” These programs include basic recommendations such as “speaking softly and in low tones,” “wearing soft shoes,” “not using incubator tops as a table surface,” “careful closing of porthole doors,” “responding promptly to alarms,” and “limiting the use of personal radios.” These behavior patterns can be supported by implementation of noise-sensor light alarms in the NICU (Chang et al., 2006). Almadhoob and Ohlsson (2015) have published further solutions for sound reduction management in a systematic literature review.

## Limitations

The recordings in the simulation room are evidently shaped by its dimension and by the placement of the speakers and microphones. Our setup was as realistic as possible and actually used devices that are employed in real life settings. However, different NICUs may have different noise surroundings dependent on room sizes and equipment. Vibrotactile information was not provided as well as binaural information from the artificial head due to massive recording artifact. A further limitation is the lack of SPL measurements for all recordings. Therefore, only relative amplitudes could be used for spectral analysis.

## Conclusion

The results of this study show a protective effect of an incubator from noises in the middle-high range as well as a boost of low frequencies within an incubator, almost no acoustic protective effect from an incubator cover, an increase of higher frequency noises with open access doors, as well as high noise levels generated by a respiratory support device widely used in the NICU setting. At maximum flow rates, the respiratory support device used in this study resulted in a significant increase in sound pressure levels inside the incubator, with the effect that noises and voices at normal listening levels became almost incomprehensible. We, therefore suggest, following the recommendations of the America Association of Pediatric (AAP) and the NAEP, the modifications of the NICU physical environment when possible, education of medical personnel and family members, as well as an acoustical optimization (damping) of the respiratory flow device (especially on high flow rates). Supplementary Audio and audiovisual materials from this study are provided, including an interactive 360° VR application to give an immersive experience of the sound environment within an incubator. Our sound examples present important opportunities for future staff training and education. Given the rhythm and the intensity of the workload within a NICU, the focus of attention can be easily switched away from noise exposure when it comes to face life-threatening situations. We believe, therefore, that awareness achieved by regular medical staff training is crucial to optimize sound quality within a NICU setting.

## Data Availability Statement

The sound files used for analysis in this study and further Supplementary Material for demonstration purposes can be found in the folder “Sound of Silence” of the Project “IncubatorExperience” at the “mdw Repository” Server of the University of Music and Performing Arts Vienna. DOI: https://doi.org/10.21939/incubator_experience.

## Author Contributions

MB and VG contributed to design of the experiment and accomplished experiment. MB, IC-E, and CR contributed to data analysis. MB, VG, CR, LB-D, AB, MO, and IC-E contributed to writing of the report. CR and MB contributed to 360°/3D recordings and VR programming.

## Conflict of Interest

The authors declare that the research was conducted in the absence of any commercial or financial relationships that could be construed as a potential conflict of interest.
