# Supplementary material for: The “Sound of Silence” in a Neonatal Intensive Care Unit—Listening to Speech and Music Inside an Incubator
Source: Front Psychol. 2020 May 26;11:1055. doi: 10.3389/fpsyg.2020.01055 (PMC7264369; doi:10.3389/fpsyg.2020.01055)
Supplement: Supplementary file 1 [file Data_Sheet_1.pdf]

Frontiers in Psychology - section Performance Science: International Symposium on Performance Science 2019
Editors: Aaron Williamon, Peter John Miksza, Masanobu Miura, Adina Mornell, Emery Schubert

The "Sound of Silence" in a neonatal intensive care unit - Listening to speech and music inside an incubator

Matthias Bertsch, Christoph Reuter, Isabella Czedik-Eysenberg, Angelika Berger, Monika Olischar, Lisa Bartha-Doering, Vito Giordano

Front. Psychol. 11:1055. doi: 10.3389/fpsyg.2020.01055 doi: 10.3389/fpsyg.2020.01055

Supplementary Material and Data
at the “mdw Repository” Server of the University of Music and Performing Arts Vienna.
Project “IncubatorExperience"
Folder "Sound of Silence”
DOI: https://doi.org/10.21939/incubator_experience

=== Sound Stimuli ===

- Acoustic stimulus 1: Broadband noise with decreasing sound level in ten steps of 6 dB
- Acoustic stimulus 2: Sine wave signals at different frequencies in ten decreasing steps of 5 dB each (125, 250, 500, 1000, 2000, 4000 and 8000 Hz)
- Acoustic stimulus 3: Sine wave signals (62.5, 125, 250, 500, 1000, 2000, 4000, 8000 and 16000 Hz)
- Acoustic stimulus 4: Logarithmic sweep (chirp) over the frequency band from 20 Hz to 21 kHz with a duration of 15 seconds
- Acoustic stimulus 5: Male voice singing
- Acoustic stimulus 6: Female voice singing and speaking
- Acoustic stimulus 7: Female voice softly singing / whispering a children’s song
- Acoustic stimulus 8: White noise

=== Audio Filenames ===
For each stimulus “x” there are 13 Sound files

- stimulus x REC 00 (co-cl-00-mic1 outside)
- stimulus x REC 01 (co-cl-00-mic2-inside)
- stimulus x REC 02 (co-cl-08-mic2-inside)
- stimulus x REC 03 (co-cl-12-mic2-inside)
- stimulus x REC 04 (uc-cl-00-mic2-inside)
- stimulus x REC 05 (uc-cl-08-mic2-inside)
- stimulus x REC 06 (uc-cl-12-mic2-inside)
- stimulus x REC 07 (uc-op-00-mic2-inside)
- stimulus x REC 08 (uc-op-08-mic2-inside)
- stimulus x REC 09 (uc-op-12-mic2-inside)
- stimulus x REF 01 (anechoic chamber-mic1)
- stimulus x REF 02 (anechoic chamber-mic2)
- stimulus x SOURCE (original digital audio)

=== Audiovisual Presentations ====

- Bertsch et al. (Sound of Silence) NICU Recording VIDEO DOCUMENTATION and examples (HD, 44.1 kHz)
- Bertsch M. (Sound of Silence) NICU Recording Stimulus 1 | Broadband noise with decreasing sound level in ten steps of 6 dB [Audiovisual Presentation]
- Bertsch M. (Sound of Silence) NICU Recording Stimulus 2 | Sine wave signals at different frequencies in ten decreasing steps of 5 dB each [Audiovisual Presentation]
- Bertsch M. (Sound of Silence) NICU Recording Stimulus 3 | Sine wave signals (62.5, 125, 250, 500, 1000, 2000, 4000, 8000 and 16000 Hz)[Audiovisual Presentation]
- Bertsch M. (Sound of Silence) NICU Recording Stimulus 4 | Logarithmic sweep (chirp) over the frequency band from 20 Hz to 21 kHz [Audiovisual Presentation]
- Bertsch M. (Sound of Silence) NICU Recording Stimulus 5 | Male voice singing [Audiovisual Presentation]
- Bertsch M. (Sound of Silence) NICU Recording Stimulus 6 | Female voice singing and speaking [Audiovisual Presentation]
- Bertsch M. (Sound of Silence) NICU Recording Stimulus 7 | Female voice softly singing & whispering a children’s song [Audiovisual Presentation]

=== The Incubator Experience (Reuter) ===

Interactive 360° VR application to give an immersive experience of the sound environment within an incubator.
Acoustic stimuli can be listened to with 360° view from inside the incubator through a Web browser (html Java).
The video can also be watched through a 3D VR headset, providing an immersive acoustic and visual demonstration of the environment within the incubator

Link: http://muwiserver.synology.me/inkubator/

=== eTable1: paired t-test across all stimuli ===

Conditions	Band 0-62 Hz	Band 62-125 Hz	Band 125-250 Hz	Band 250-500 Hz	Band 500-1000 Hz	Band 1000-2000 Hz	Band 2000-4000 Hz	Band 4000-8000 Hz	Band 8000-16000 Hz	Band 16000-20000 Hz
Outside incubator vs Inside (covered and closed)	.0001	.0004	.0078	.0178	.0003	.0001	.0003	.0003	.0001	.0307
Using of the cover [(covered-closed-inside the incubator) vs (uncovered-closed-inside the incubator)]	.1172	.0419	.0001	.0301	.0414	.5387	.0001	.0032	.0004	.1630
Incubator doors[(closed-uncovered) vs (open-uncovered)]	.0105	.1527	.0001	.0001	.0001	.0005	.0008	.0003	.0004	.0065
Oxygen support [(Flow00_inside)_vs_(Flow12_inside)]	9070	.1747	.2042	.8045	.0001	.0001	.0001	.0001	.0001	.0001
Oxygen support [(Flow00_inside)_vs_(Flow08_inside)]	.6896	.1474	.3201	.0134	.0001	.0001	.0001	.0001	.0001	.0001
Oxygen support [(Flow12_inside)_vs_(Flow08_inside)]	.2943	.7893	.7171	.0015	.0010	.0001	.0001	.0001	.0001	.0002
